# A J-aggregated nanoporphyrin overcoming phototoxic side effects in superior phototherapy with two-pronged effects†

**DOI:** 10.1039/d2sc04873f

**Published:** 2022-10-15

**Authors:** Mengyao Yang, Xingshu Li, Gyoungmi Kim, Rui Wang, Seong-Jin Hong, Chang-Hee Lee, Juyoung Yoon

**Affiliations:** Department of Chemistry and Nanoscience, Ewha Womans University Seoul 03760 Republic of Korea jyoon@ewha.ac.kr; College of Chemistry, State Key Laboratory of Photocatalysis on Energy and Environment, Fujian Provincial Key Laboratory of Cancer Metastasis Chemoprevention and Chemotherapy, Fuzhou University Fuzhou 350108 China xingshuli@fzu.edu.cn; Department of Chemistry and Biochemistry, Kangwon National University Chun Cheon 24341 Republic of Korea chhlee@kangwon.ac.kr

## Abstract

Phototherapy has been a promising therapeutic modality for pathological tissue due to its spatiotemporal selectivity and non-invasive characteristics. However, as a core component of phototherapy, a single photosensitizer (PS) nanoplatform integrating excellent therapeutic efficiency and minimal side effects remains an urgent but unmet need. Here, we construct a J-aggregated nano-porphyrin termed MTE based on the self-assembly of methyl-pheophorbide a derivative MPa-TEG (MT) and natural polyphenolic compound epigallocatechin gallate (EGCG). Due to the synergistic interaction between similar large π-conjugated structural EGCG and MT, MTE with small and uniform size is obtained by effectively hindering Ostwald ripening of MT. Noteworthily, MTE not only effectively avoids the inadvertent side effects of phototoxicity during transport thank to the ability of reactive oxygen species (ROS) scavenging, but also achieves two-pathway augmented superior phototherapy: (1) enhancing photodynamic therapy (PDT) *via* inhibiting the expression of anti-apoptosis protein surviving; (2) achieving adjuvant mild-temperature laser interstitial thermal therapy (LITT) *via* reducing the tumor thermoresistance on account that MTE inhibits the overexpression of HSP 70 and HSP 90. This research not only offers a facile strategy to construct multicomponent nanoplatforms but also provides a new pathway for efficient and low-toxicity phototherapy, which is beneficial to the future clinical application.

## Abstract 2

J-aggregated nanoporphyrin (MTE) integrates minimal side effects and two-pathway augmented superior phototherapy: enhancing photodynamic therapy (PDT) and achieving adjuvant mild-temperature laser interstitial thermal therapy (LITT).

## Graphical abstract

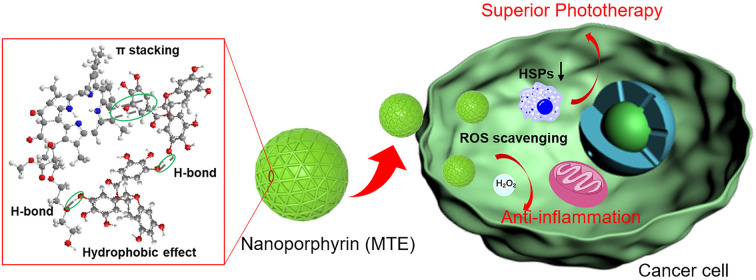

## Introduction

Phototherapy, which is defined as a treatment triggered by photons, has been a promising modality for neoplasms due to its unique features, including less invasiveness, minimal drug resistance, and selective spatiotemporal distribution.^1–5^ Photodynamic therapy (PDT) and photothermal therapy (PTT) utilize photosensitizers (PSs) to generate reactive oxygen species (ROS) and heat, respectively, under the irradiation of appropriate wavelengths of light to cause selective damage to tumors or lesions.^6–8^ In particular, laser interstitial thermal therapy (LITT) without exogenous PSs has been clinically applied for hyperthermia ablation.^9,10^ Although a large number of phototherapy studies have been conducted, most of them suffer from unsatisfactory therapeutic effects and serious side effects. The prevailing dilemma of PDT efficacy is the “Achilles' heel” of hypoxia in the tumor environment, which has been the research hotspot for overcoming the bottleneck of PDT efficacy. For example, some emerging PSs effectively avoid oxygen dependence *via* a type I mechanism,^11–14^ and others have utilized supplemental oxygen to treat this challenging problem.^15,16^ Most of the constructed PSs are mainly based on methods to increase ROS generation and subsequently improve the therapeutic effect; nevertheless, the expression of antiapoptotic client proteins during PDT has often been overlooked.

Heat shock proteins (HSPs) are overexpressed in response to external stimuli, which not only impede PTT due to heat resistance^17,18^ but also substantially reduce the efficiency of PDT because the antiapoptotic protein survivin, as a client protein for HSPs, blocks apoptosis induced by PDT through the inhibition of caspase-9.^19,20^ Therefore, a local temperature higher than 50 °C is required for PTT to completely kill tumor cells, while increases in the power and time of light irradiation are needed for PDT to achieve good efficiency, inevitably causing damage to the surrounding normal tissues and cells.^21–23^ By circumventing the conventional methods of increasing the efficiency of photothermal conversion or increasing ROS production, the inhibition of HPSs overexpression overcomes tumor thermoresistance and inhibits the production of antiapoptotic client proteins during PDT to enhance the final therapeutic effect from the source, which will be a great step forward in the clinical application of phototherapy.

Since porphyrin-based PSs have a very strong absorption band at approximately 400 nm called the Soret band due to the large ring conjugation structure of 18 π-electrons,^24,25^ they hardly avoid damaging nonfocal tissues through inadvertent ROS generation induced by natural light in the nontherapeutic stage.^26^ Therefore, in the clinical application of PDT, patients must remain in the dark environment for a long time, which not only prohibits the action of the patient but also increases obstacles to the treatment process, substantially hampering its clinical application. Additionally, the excitation wavelength of light used for porphyrin-based PSs is typically 600–650 nm in the Q band, limiting the depth of tissue penetration.^27–29^ Thus, the substructural stacking of J-aggregates in nanoengineering, which leads to redshifted absorption, will be a breakthrough to solve this problem.^30–32^

**Scheme 1:**
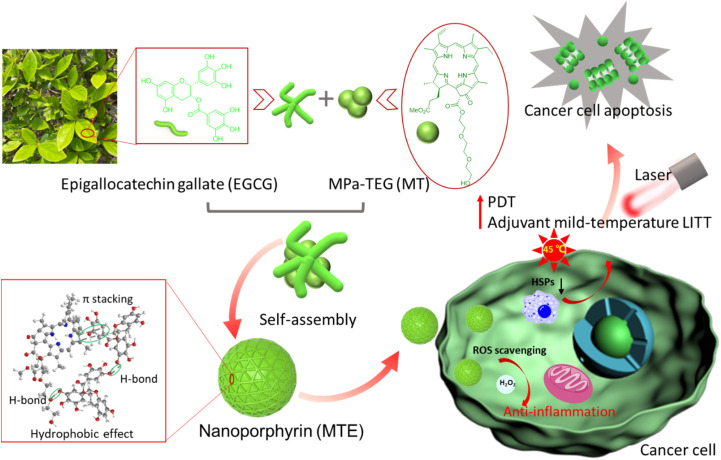
Schematic illustration of J-aggregated nanoporphyrin (MTE) based on natural EGCG and MT for excellent antitumor PDT and adjuvant mild-thermal LITT. Due to the antioxidant effect of EGCG and its potential as an inhibitor of HSPs, MTE has a good ability to scavenge ROS and inhibit the expression of HSPs, achieving safe and excellent PDT and adjuvant mild-temperature LITT.

Inspired by mussels with magical adhesion effects due to functional unit catechins,^33–35^ herein, a J-aggregated nanoporphyrin termed MTE based on the self-assembly of triethylene glycol decorated methyl-pheophorbide a MPa-TEG (MT) and natural polyphenolic compound epigallocatechin gallate (EGCG) was obtained through multiple noncovalent hydrogen bonding, π–π stacking and hydrophobic interactions. Due to the strong hydrophilicity of EGCG and hydrophobicity of MT causing by the large conjugated ring, EGCG mainly covered the surface of MT, and MTE with a small and uniform size was obtained by effectively hindering the formation of irregular aggregates and precipitation caused by Ostwald ripening of MT (Scheme 1). In addition, the absorption redshift of the J-aggregate is favorable for a greater tissue penetration depth of light, and the J-aggregate remains stable even in biological environments. Notably, MTE retains the outstanding properties of ROS scavenging and HSP inhibition. The inhibition of HSP overexpression induced by irradiation not only reduces tumor thermoresistance but also suppresses the expression of survivin, increasing the efficiency of PDT by activating the apoptosis pathway. ROS scavenging not only eliminates the side effects of phototoxicity caused by nontarget activity and inevitable visible light but also eliminates the inflammation and damage to normal tissues caused by excessive intracellular ROS accumulation. These processes exert a two-pronged effect, achieving two-stage augmented safe and superior PDT and adjuvant mild-temperature LITT. To the best of our knowledge, this study is the first to utilize EGCG as an HSP inhibitor and ROS scavenger for PDT and adjuvant mild-temperature LITT, which provides not only a facile protocol to construct nanoplatforms but also a new pathway for efficient and low-toxic phototherapy. This study is of great significance for future clinical applications.

## Results and discussion

### Design of MTE

**Fig. 1:**
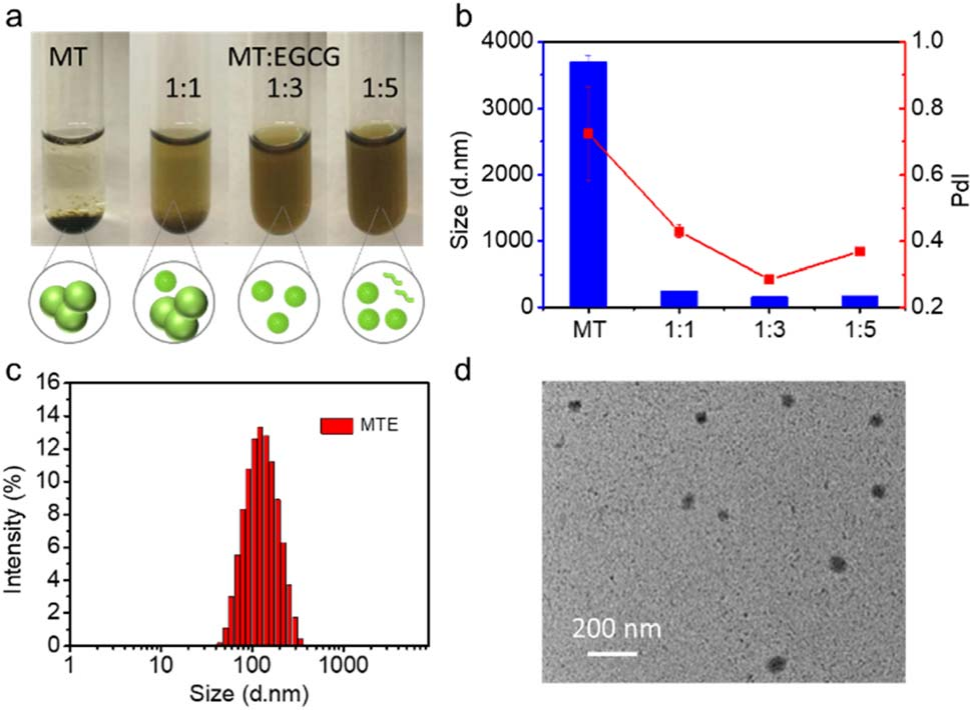
The preparation and characterization of MTE. (a) The optical pictures and schematic illustration of different feed ratios of MT and EGCG. (b) DLS profiles of MTE (100 μM) with different ratios of MT-EGCG (MT: single MT, 1 : 1 is MT : EGCG = 1 : 1, 1 : 3 is MT : EGCG = 1 : 3, 1 : 5 is MT : EGCG = 1 : 5). (c) DLS profile of MTE (10 μM). (d) TEM images of MTE (10 μM).

EGCG, a major natural component of green tea, has been a popular building block for constructing bionanomaterials due to its excellent biocompatibility and biological activity.^36^ In particular, EGCG has shown a promising potential as an inhibitor of HSPs,^37–39^ which provides good inspiration to construct nano-PSs for safe and effective phototherapy. In addition, the presence of a large number of hydroxyl groups provides multiple sites for noncovalent interactions.^36^ Meanwhile, due to the superior optical properties of porphyrin-based PSs, MT is preferred as the core photosensitizer, in which the triethylene glycol is conjugated with methyl pheophorbide a (MPa), as shown in Schemes 1 and S1.† The introduction of triethylene glycol not only increases the hydrophilicity of MT but also increases the binding sites for the formation of hydrogen bonds with EGCG, facilitating the formation of nanocomplexes. The structure of MT was verified by high-resolution electrospray ionization mass spectroscopy (HR-ESI-MS) and nuclear magnetic resonance (^1^H/^13^C NMR) characterizations shown in Fig. S1–S3.† Fortunately, the structural regulation allows MT to exhibit a greater redshift and a higher extinction coefficient in aqueous solution than MPa (Fig. S4†). When the solvent containing MT was added to the aqueous solution containing EGCG, the MT rapidly nucleated and was uniformly distributed under the assistance of ultrasound. At the same time, the synergistic interaction between similar large π-conjugated structural EGCG and MT was facilitated to form the stable nanocomposite (MTE). Compared to the strong aggregation behavior of individual MT molecules (Fig. 1a), the presence of EGCG effectively inhibited the aggregation and precipitation caused by Ostwald maturation of MT.^40^

### Preparation and characterization of MTE

The feed ratio of EGCG and MT was adjusted to obtain small and uniform nanocomplexes. The optical images showed substantial aggregation and precipitation in an aqueous solution of MT alone due to its strong hydrophobicity, while the precipitate disappeared with increasing EGCG concentrations (Fig. 1a), revealing that the presence of EGCG effectively prevented the Ostwald ripening of MT. The dynamic light scattering (DLS) results also revealed that MT alone in aqueous solution exhibited substantial aggregation, while the addition of EGCG contributed to the formation of nanocomplexes. The ratio of 1 : 3 MT : EGCG, termed MTE, with a zeta potential of −18.57 ± 0.35 mV was selected for further application (Fig. 1b) since it had a narrow size distribution with the best polydispersity index (PdI: 0.284), and the size was approximately 150 nm. The addition of higher EGCG concentrations may lead to the presence of free EGCG molecules, affecting the polydispersity index (Fig. 1a). Fig. 1c shows a size of MTE of approximately 150 nm after ten-fold dilution. This result was consistent with the transmission electron microscopy (TEM) images (Fig. 1d), implying that MTE had good resistance to dilution. In addition, the size of MTE showed almost no discernible change even after one week (Fig. S5†). J-aggregation is usually interrupted in the biological environment.^41^ Different concentrations of fetal bovine serum (FBS) were incubated with MT and MTE separately to examine the stability of J-aggregates under physiological conditions. The Q band of MT split into two peaks at around 670 and 690 nm after incubating with FBS. As the concentration of FBS increased, the intensity of the peak at around 670 nm increased and the peak at 690 nm correspondingly decreased, which was because MT gradually became a monomer (Fig. S6†). In contrast, the absorption band of MTE did not alter, reflecting MTE maintained stable J-aggregation even after incubation with 90% FBS (Fig. S7†). Intriguingly, while maintaining J-aggregate stability, the fluorescence of MTE was intelligently enhanced by FBS (Fig. S8†), which is beneficial for biological imaging.

### Self-assembly mechanism of MTE

**Fig. 2:**
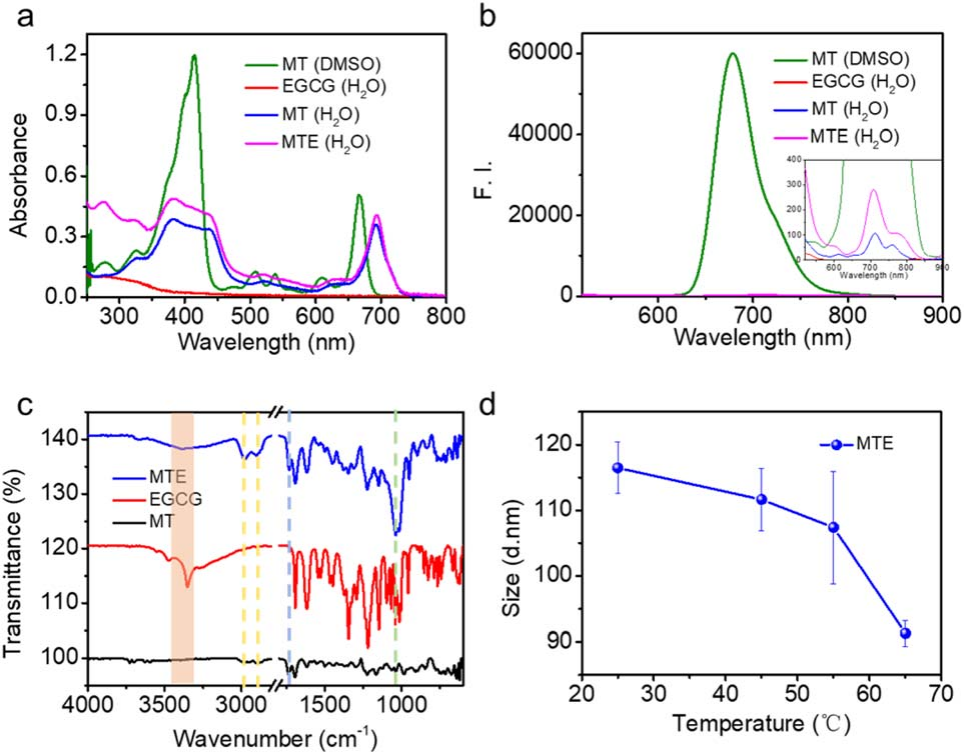
The mechanism of MTE self-assembly. (a) Electronic absorption, (b) fluorescence, and (c) FT-IR spectra of MTE and monomeric MT and EGCG. (d) DLS profiles of MTE at different temperatures.

To investigate the MTE self-assembly mechanism, electronic absorption, fluorescence, Fourier transform infrared (FT-IR) spectroscopy, and DLS at various temperatures of MTE were measured. Electronic absorption showed that compared with MT monomer molecules, the assemblies underwent a significant redshift, implying the formation of J-aggregates (Fig. 2a), which enhanced the penetration of the tissue due to the near-infrared (NIR) biological absorption window. This property is beneficial for biomedical applications. Additionally, fluorescence quenching indicated the existence of π–π stacking and hydrophobic interactions between the benzene ring and porphyrin ring (Fig. 2b). Four characteristic bands were observed in the FT-IR spectrum of MTE (Fig. 2c). The band at 3350 cm^−1^ is assigned to the stretching vibration of the O–H group, which underwent a significant redshift and was weakened compared with the corresponding band of EGCG. The band at 1733 cm^−1^ is due to the stretching vibration absorption of C=O, whose relative decrease in peak strength might also be attributed to the formation of hydrogen bonds between the O–H groups of EGCG and MT. The bands at 2981 and 2895 cm^−1^ correspond to amide N–H stretching. All of these results implied that the formation of MTEs was coordinated by MT and EGCG. Moreover, the band at 1030 cm^−1^ is assigned to C–O, which was also enhanced compared with the corresponding bands in the FT-IR spectra of MT and EGCG due to the successful coassembly.^42^ The DLS profile of MTE at varying temperatures was tested to further verify the hydrogen bonds (Fig. 2d). The size of MTE decreased with increasing temperature and was attributed to the destruction of hydrogen bonds between MT and EGCG,^43,44^ causing partial disassembly of EGCG molecules. This indicates that the self-assembly process was mainly mediated by hydrogen bonding between molecules.

### Photochemical and ROS-scavenging performances of MTE

**Fig. 3:**
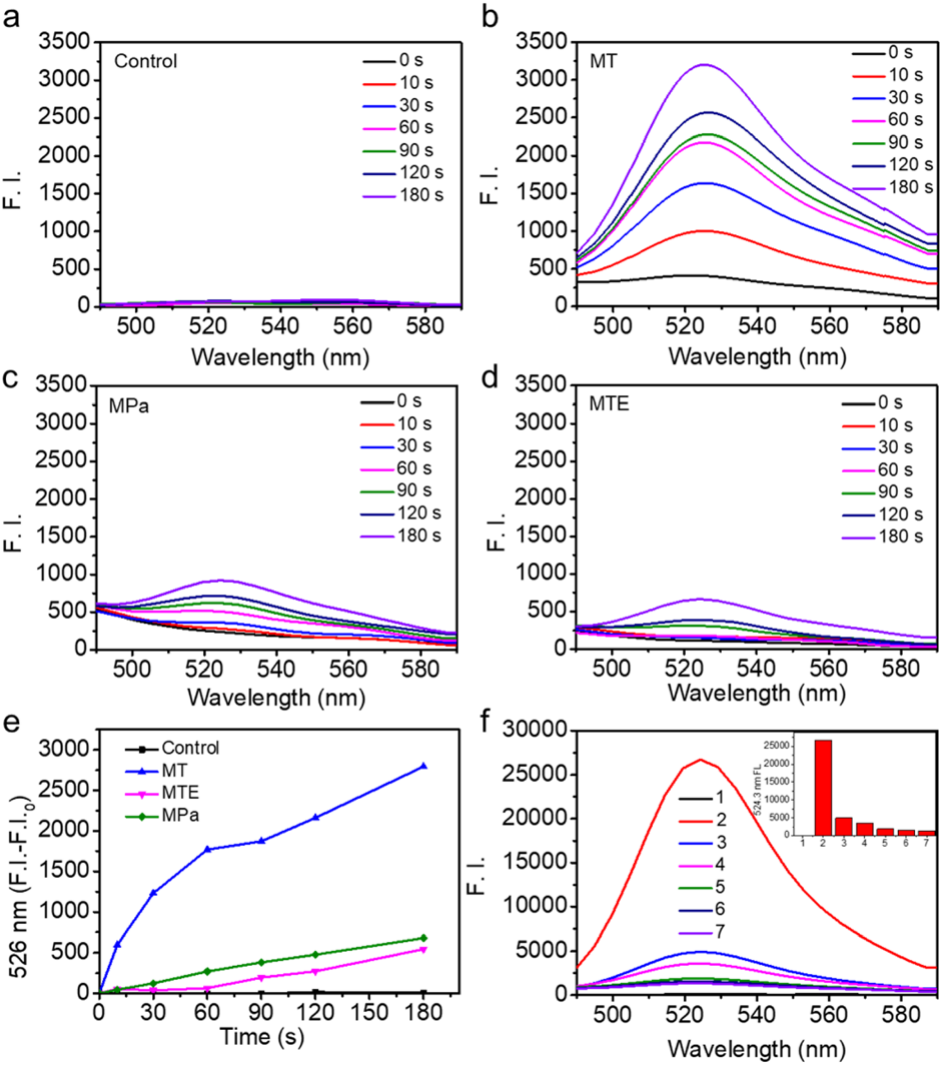
Photochemical and ROS-scavenging performances of MTE. ROS generation by the (a) control, (b) MT (c) MPa and (d) MTE (all of them are 10 μM) in water solutions using DCFHDA as a probe. (e) The change in fluorescence intensity at 526 nm with irradiation time (690 nm laser, 0.1 W cm^−2^). (f) ROS-scavenging ability of MTE using DCFHDA as the fluorescence probe (the inset shows the fluorescence intensity, (1) only DCFHDA, (2) H_2_O_2_ + DCFHDA, (3) H_2_O_2_ + 10 μM MTE + DCFHDA, (4) H_2_O_2_ + 20 μM MTE + DCFHDA, (5) H_2_O_2_ + 30 μM MTE + DCFHDA, (6) H_2_O_2_ + 40 μM MTE + DCFHDA, (7) H_2_O_2_ + 50 μM MTE + DCFHDA).

The potential of MTE for phototherapy was evaluated by determining its photochemical performance. First, we tested the ROS generation induced by different PSs using 2,7-dichlorofluorescin diacetate (DCFHDA) as a probe and the group containing only DCFHDA as the control (Fig. 3a). MT exhibited an excellent ROS production compared with MPa (Fig. 3b and c). This was mainly due to the increase in the extinction coefficient of MT mediated by the structural modification, which was more favorable for the excitation of PSs to enhance ROS generation. Furthermore, MTE produced less ROS than MT (Fig. 3d and e), which was mainly attributed to the aggregation mode leading to a change in the pathway of energy decay and the ROS-scavenging ability of EGCG causing by multiple active phenolic hydroxyl groups.^45,46^ Different concentrations of MTE solution were coincubated with H_2_O_2_ using DCFHDA as the probe to verify the ROS-scavenging ability (Fig. 3f). The fluorescence intensity decreased with increasing MTE concentrations, demonstrating that MTE had an admirable ability to scavenge ROS. Because of the Soret band absorbance peak at approximately 400 nm, the clinical PSs based on porphyrins inevitably induce phototoxicity when exposed to visible light. Thus, ROS-scavenging is important because it not only effectively avoids the inadvertent phototoxicity induced by visible light in nontreatment times but also has the potential against inflammatory effects induced by ROS. Abnormal metabolism of carcinomatous cells and tissues results in excess ROS production, thus inducing inflammation and damage to normal tissues and further limiting treatment.^46,47^ Therefore, the elimination of inflammation is a key factor contributing to the effective treatment of cancers.

### Cellular uptake of MTE and cell viability assay

**Fig. 4:**
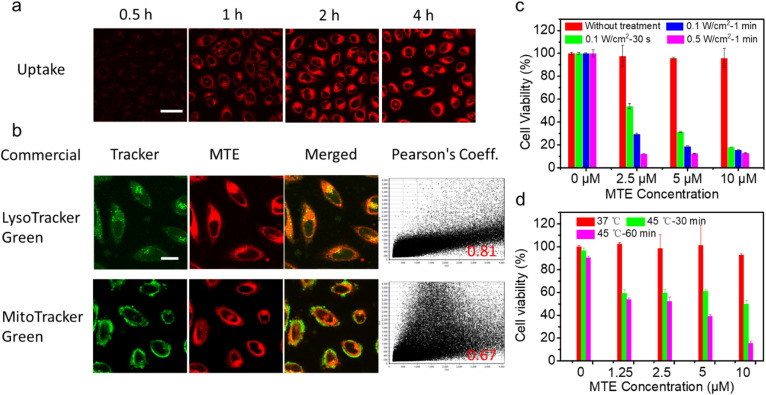
(a) CLSM images of the cellular uptake of MTE in HeLa cells. Scale bar: 50 μm. (b) CLSM images showing the colocalization of MTE with a commercial tracker in HeLa cells. Scale bar: 20 μm. The viability of HeLa cells treated with different concentrations of MTE (c) and irradiated with a 690 nm laser. (d) Cells were incubated in a 45 °C water bath to evaluate tumor thermal resistance.

Encouraged by the excellent photo-properties and ROS-scavenging ability of MTE, we conducted cell-based experiments. First, the cellular uptake of MTE in Human cervical carcinoma cells (HeLa cells) was observed using confocal laser scanning microscopy (CLSM) (Fig. 4a). The fluorescence of MT increased gradually with a prolonged incubation time, indicating that MTE was effectively internalized into the cytoplasm, mainly due to the entry of MTE into cells through endocytosis. Commercial probes LysoTracker Green (LTG) and MitoTracker Green (MTG) were co-stained with MTE in HeLa cells to further examine the location of MTE (Fig. 4b). MTE was mainly distributed in lysosomes, with a high Pearson's correlation coefficient (0.81). Noteworthy, the CLSM results using DCFHDA as the fluorescent ROS probe show that MTE effectively avoided inadvertent ROS generation induced by visible light compared with MT (Fig. S9†). In addition, the introduction of triethylene glycol in MPa substantially increased the production of ROS in the cellular environment. A typical 3-(4,5-dimethylthiazol-2-yl)-2,5-diphenyltetrazolium bromide (MTT) assay was conducted to further assess the PDT efficacy of MTE in cancer cells (Fig. 4c). HeLa cells were cocultured with various concentrations of MTE for 2 h and then treated with different conditions after replacement with new medium. HeLa cells treated with MTE in the absence of irradiation displayed negligible cytotoxicity, reflecting the good biocompatibility of MTE. In addition, cell viability was reduced by MTE after irradiation, and the inhibitory efficiency depended on the MTE concentration and laser power. Notably, 18.16 ± 0.11% of HeLa cells were viable after an incubation with 10 μM MTE and irradiation with 0.1 W cm^−2^ for 30 s, displaying an excellent PDT effect.

A 45 °C water bath was applied to treat HeLa cells as a method to verify whether MTE reduces tumor thermoresistance and whether it assists in achieving mild-temperature therapy. Significantly, as the concentration of MTE-treated HeLa cells increased, cell viability was effectively reduced (Fig. 4d), which might be ascribed to the reduced thermal resistance of HeLa cells. The control group not treated with MTE retained 90.33 ± 1.60% cell viability even after an incubation at 45 °C for 60 min, demonstrating the resistance of HeLa cells to heat stress. The viability of HeLa cells treated with 10 μM MTE was 15.36 ± 2.08%, potentially due to the inhibition of HSP expression by EGCG, displaying that MTE has the potential for use as a good adjuvant in mild-temperature therapy combined with exogenous heat sources, such as LITT.

### Apoptosis mechanism of the superior phototherapy mediated by MTE in HeLa cells

**Fig. 5:**
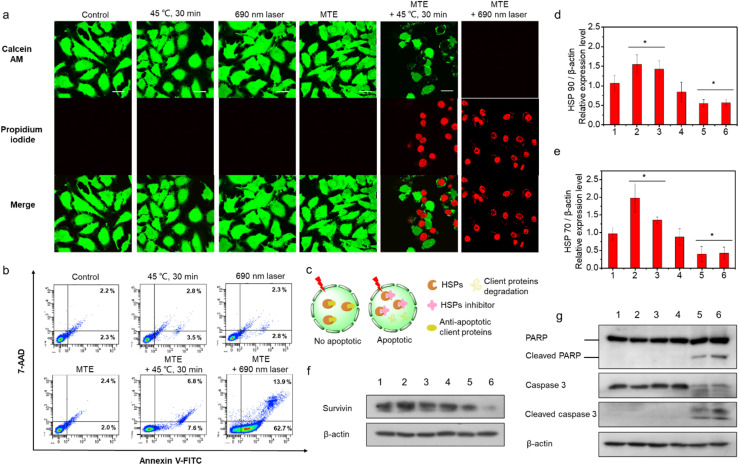
The apoptosis mechanism of the superior phototherapy mediated by MTE in HeLa cells. (a) Live/dead cell assay, where cells were stained with calcein AM and PI and fluorescence images were acquired using confocal microscopy (calcein AM: ex: 473 nm/em: 490–590 nm, PI: ex: 559 nm/em: 575–675 nm), scale bar: 30 μm. (b) Representative flow cytometry plots of cells stained with Annexin V-FITC and 7-AAD (7-aminoactinomycin D) and analyzed using flow cytometry. (c) Schematic diagram of the inhibition of apoptosis and apoptosis induced by phototherapy of carcinoma cells in the presence or absence of a heat shock protein inhibitor. Bar graph showing relative (d) HSP 90 (e) HSP 70 expression levels from three independent experiments (*n* = 3) calculated with ImageJ software. *P < 0.05, significantly different from the control. (f) Survivin protein expression level in HeLa cells. (g) Levels of PARP and caspase 3 in HeLa cells. The expression of β-actin was used as a loading control ((1) control, (2) 45 °C for 30 min, (3) 690 nm laser, (4) MTE, (5) MTE + 45 °C for 30 min, (6) MTE + 690 nm laser).

Live and dead cells were costained with calcein AM (green, live cells) and propidium iodide (PI) (red, dead cells) (Fig. 5a) to further confirm the two-pathway augmented superior phototherapy, including superior PDT of MTE and whether MTE achieved adjuvant mid-thermal therapy combined with exogenous heat energy (such as LITT). HeLa cells were incubated with or without MTE (3 μM) for 2 h and placed in a 45 °C water bath for 30 min or irradiated with a 690 nm laser (0.1 W cm^−2^) to test the effect of MTE on promoting mild thermal therapy and the superior PDT of MTE. A substantial number of HeLa cells pretreated with MTE died after mild hyperthermia and light irradiation treatment compared with their counterparts. A flow cytometry analysis was performed to obtain additional insights into the cell apoptosis rate (Fig. 5b and S10†). The results clearly show that compared with the control groups, HeLa cells pretreated with MTE exhibited higher cell apoptosis rates after treatment at 45 °C for 30 min, and the apoptosis rate (early + late apoptosis) of HeLa cells treated with MTE and irradiated with a 690 nm laser was as high as 78.5%, indicating that cell death was mainly mediated by apoptosis pathways. As a proof-of-concept study, EGCG was assumed to function as an inhibitor of HSPs (Fig. 5c). Under normal circumstances, cells overexpress HSPs to resist heat damage when exposed to external stimuli. At the same time, HSPs also bind some antiapoptotic proteins to block cell apoptosis. Survivin, a client protein of HSPs, inhibits apoptosis induced by PDT. When HSPs expression is reduced, the thermal resistance of cancer cells is reduced and the production of antiapoptotic proteins during PDT is also eliminated, thus achieving an outstanding therapeutic effect through both mechanisms. Thus, Western blot analysis was performed to explore the mechanism of efficient cell apoptosis. The HSPs HSP 70 and HSP 90 were expressed at higher levels in the HeLa cells treated with heat or light irradiation than in the untreated control cells (Fig. S11† and 5d, e). In contrast, MTE effectively inhibited the overexpression of HSP 70 and HSP 90 induced by mild hyperthermia and light irradiation in HeLa cells, consistent with the hypothesis. Moreover, the expression of the antiapoptotic protein survivin was increased when HeLa cells were treated with 45 °C incubation or light irradiation, while it was inhibited effectively by MTE, which strongly confirmed our speculation (Fig. 5f and S12†). Furthermore, as the key effectors of apoptosis, levels of cleaved PARP, cleaved caspase-3 and cleaved caspase-9 were detected to assess the apoptosis pathway (Fig. 5g, S13 and S14†).^48,49^ The increased levels of these proteins further confirmed that HeLa cell apoptosis was inhibited by HSP expression, while MTE accelerated cell apoptosis by eliminating the inhibitory effect on apoptosis.

### ROS-scavenging activity of MTE in cells

**Fig. 6:**
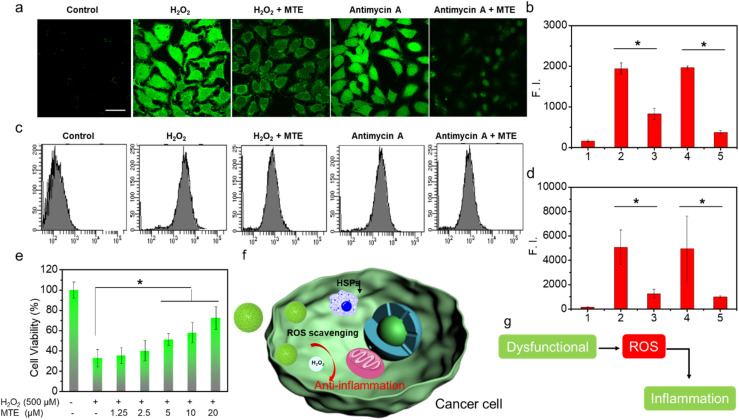
ROS scavenging assay. (a) Fluorescence images were obtained using confocal microscopy (DCFHDA: ex. 473 nm/em. 490–590 nm). Scale bar: 30 μm. (c) Flow cytometry data were obtained using an LSRFortessa instrument. Quantitative fluorescence intensity in (b) CLSM images, (d) flow cytometry data showing the amount of residual ROS. (e) The viability of HeLa cells treated with H_2_O_2_ ± various concentrations of MTE. Schematic diagram of (f) anti-inflammation around tumors. (g) Relationship between ROS and inflammation. Data are presented as the means ± standard deviations from three independent experiments. *P < 0.05, significantly different by comparing two groups.

MTE exhibits good ROS-scavenging activity *in vitro*, which potentially avoids the cytotoxicity caused by inevitable exposure to visible light in the nontherapeutic stage. In addition, ROS overproduction is often associated with tumors and other lesions by inducing inflammation.^50–52^ If the nano-PSs/drugs themselves exert an anti-inflammatory effect during the treatment process, they will not only facilitate the treatment of cancer but also protect normal cells from the damage induced by inflammatory factors. As methods to obtain additional insights into the ROS-scavenging ability, fluorescence images captured using CLSM and flow cytometry data obtained using LSRFortessa were analyzed to determine the ROS-scavenging ability at the cellular level through imaging the intracellular ROS level (Fig. 6). HeLa cells incubated with H_2_O_2_ exhibited bright green fluorescence (Fig. 6a and b). In contrast, HeLa cells preincubated with MTE and then treated with H_2_O_2_ showed negligible ROS levels, displaying the ROS-scavenging ability of MTE. In addition, antimycin A has been shown to generate ROS; thus, HeLa cells treated with antimycin A also displayed bright green fluorescence. The ROS level was significantly reduced by MTE. This result is consistent with the results of the flow cytometry analysis (Fig. 6c and d). Similar results further confirmed that MTE effectively eliminated excess ROS from the cell, which effectively avoided the damage caused by excess ROS in cells, such as inflammation, and reduced the side effects during the treatment process. Furthermore, the cell viability measured by MTT assay verified that MTE exerted a concentration-dependent effect on mitigating cell damage induced by H_2_O_2_ (Fig. 6e). H_2_O_2_ has been known to cause severe inflammation by inducing the production of significant levels of tumor necrosis factor α (TNF-α) in macrophages, suggesting that MTE has anti-inflammatory potential.^50–52^ Several diseases utilize higher intracellular ROS levels as a vital factor to promote progression.^53,54^ Abnormal metabolism in tumors leads to the excessive accumulation of ROS, leading to inflammation around tumors (Fig. 6f and g), which is often one of the key points impeding clinical treatment. Therefore, ROS elimination is particularly important when treating pathologies of cancer and other related diseases.

## Conclusions

In summary, we have first successfully developed a nanoporphyrin (MTE) based on MT and EGCG through the synergistic effects of hydrogen bonding, π–π stacking and hydrophobic interactions between aromatic MT and EGCG. As a proof-of-concept study that EGCG is a potential HSP inhibitor, MTE inhibited the overexpression of HSP70 and HSP90 induced by external stimuli and thus eliminated heat resistance to achieve adjuvant mid-thermal therapy combined with exogenous heat energy. The expression of survivin, a client protein for HSPs, was also reduced and thus relieved its inhibitory effect on PDT apoptosis, improving PDT efficiency *via* the apoptosis pathway. Therefore, MTE achieved safe and efficacious PDT and adjuvant mild-temperature LITT. In addition, MTE exhibited good scavenging of ROS, which not only reduced the side effects caused by inevitable exposure to visible light in the phototherapy process but also exerted a potential anti-inflammatory effect, increasing treatment efficacy. Overall, MTE exhibits minimal side effects, and it targets two pathways to augment superior PDT and adjuvant mild-temperature LITT by substantially reducing tumor thermoresistance and suppressing survivin expression. This research not only provides a facile proposal to fabricate multifunctional nanoplatforms but also a novel pathway for the development of efficient and low-toxic phototherapy, which would be a great impetus for future clinical applications.

## Data availability

The datasets supporting this article have been uploaded as part of the ESI material.†

## Author contributions

Mengyao Yang: conceptualization, formal analysis, data curation, visualization, writing – original draft; Xingshu Li: conceptualization, supervision, writing – review & editing; Gyoungmi Kim: data curation, methodology, investigation; Rui Wang: formal analysis, data curation; Seong-Jin Hong: methodology, formal analysis; Chang-Hee Lee: resources, funding acquisition; Juyoung Yoon: conceptualization, supervision, project administration, funding acquisition.

## Conflicts of interest

The authors declare no competing financial interests.

## Supplementary Material

SC-013-D2SC04873F-s001

SC-013-D2SC04873F-s002
