# Supplementary material for: A J-aggregated nanoporphyrin overcoming phototoxic side effects in superior phototherapy with two-pronged effects
Source: Chem Sci. 2022 Oct 15;13(43):12738–46. doi: 10.1039/d2sc04873f (PMC9645379; doi:10.1039/d2sc04873f)

Electronic Supplementary Material (ESI) for Chemical Science.
This journal is © The Royal Society of Chemistry 2022

Supporting information 2:

1. Full gel and blot for Fig. S11 (HSP90 and HSP70)

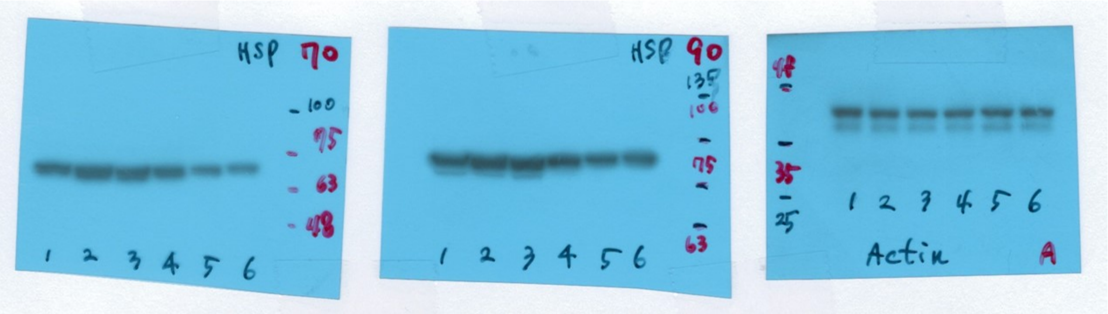

2. Full gel and blot for Fig. 5f (survivin) and Fig. S14 (caspase 9)

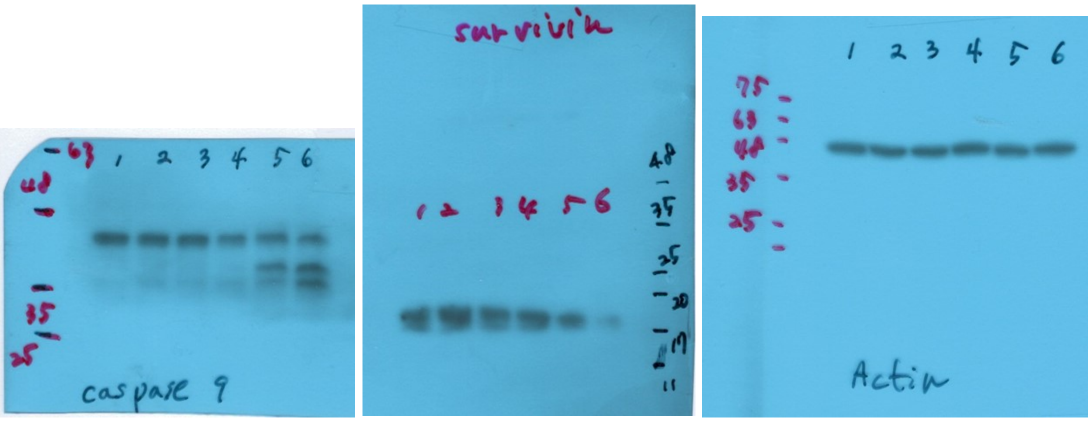

(1. control, 2. 45 °C for 30 min, 3. 690 nm laser, 4. MTE, 5. MTE + 45 °C for 30 min, 6. MTE + 690 nm laser)

3. Full gel and blot for Fig. 5g (caspase 3 and PARP)

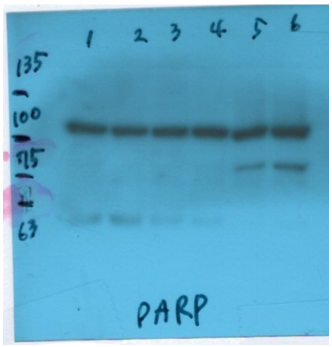

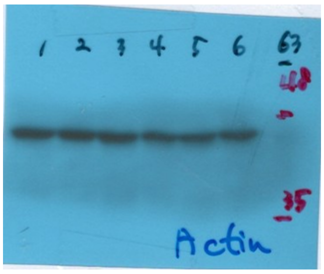

Caspase 3

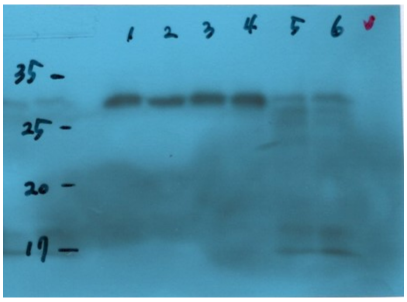

Cleaved Caspase 3

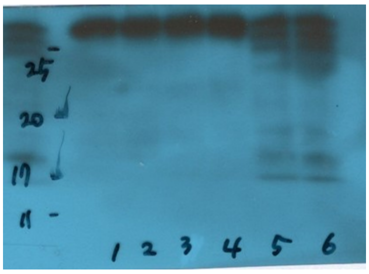

Supplement: SC-013-D2SC04873F-s001 [file SC-013-D2SC04873F-s001.pdf]
