# Supplementary material for: A J-aggregated nanoporphyrin overcoming phototoxic side effects in superior phototherapy with two-pronged effects
Source: Chem Sci. 2022 Oct 15;13(43):12738–46. doi: 10.1039/d2sc04873f (PMC9645379; doi:10.1039/d2sc04873f)
Supplement: SC-013-D2SC04873F-s002 [file SC-013-D2SC04873F-s002.pdf]

Electronic Supplementary Material (ESI) for Chemical Science.
This journal is © The Royal Society of Chemistry 2022

Supporting Information for:

A J-aggregated Nanoporphyrin Overcoming Phototoxic Side Effects in Superior Phototherapy with Two-Pronged Effects

Mengyao Yang,[a] Xingshu Li,*[b] Gyoungmi Kim,[a] Rui Wang,[a] Seong-Jin Hong,[c] Chang-Hee Lee,*[c] Juyoung Yoon*[a]

[a] Department of Chemistry and Nanoscience, Ewha Womans University, Seoul 03760, Republic of Korea.

[b] College of Chemistry, State Key Laboratory of Photocatalysis on Energy and Environment, Fujian Provincial Key Laboratory of Cancer Metastasis Chemoprevention and Chemotherapy, Fuzhou University, Fuzhou 350108, China.

[c] Department of Chemistry and Biochemistry, Kangwon National University, Chun Cheon 24341, Republic of Korea.

E-mail: jyoon@ewha.ac.kr; xingshuli@fzu.edu.cn; chhlee@kangwon.ac.kr

Table of contents

1. Experimental Section……………………………………………………......................S3
2. Scheme S1………………..………………………………………………………….S6
3. Figure S1 (HR-ESI-MS)..…..……..…………………………………………………S6
4. Figure S2 (^{1}H-NMR) …………………………………………………………..S7
5. Figure S3 (^{13}C-NMR) ………………………………..........……………………..S8
6. Figure S4………………………………………………………….……………….S8
7. Figure S5…….…………………………………….……………….……..................S9
8. Figure S6……………………………….….………………………………………....S9
9. Figure S7………………………...….…………………………………………………S10
10. Figure S8………….……………………………………………..….….................…..S10
11. Figure S9..………………………….……………...……………………………………...S10
12. Figure S10.……...S11
13. Figure S11...……………………………………………………………………....S11
14. Figure S12……………………...…...…...S11
15. Figure S13………………………………………………………………………...S12
16. Figure S14………………………………………………………………………...S12

1. Experimental Section

1.1 Materials and instruments

(-)-Epigallocatechin gallate (EGCG) was purchased from TCI corporation. Dimethylsulfoxide (DMSO) and 2,7-dichlorofluorescin diacetate (DCFHDA) were obtained from Sigma–Aldrich Korea. MitoTracker and LysoTracker were provided by Life Technologies Co. (USA). All reagents were used without further purification.

^{1}H NMR and ^{13}C NMR spectra were measured using a 300 MHz Bruker spectrometer. High resolution electrospray ionization mass spectroscopy (HR-ESI-MS) data was obtained using a SYNAPT G2 (Waters, U.K.) spectrometer at the Korea Basic Science Institute (Ochang). UV–visible absorption spectra were measured with a Thermo Scientific Evolution 201 UV–Visible spectrophotometer, and all fluorescence measurements were recorded using an FS-2 fluorescence spectrometer (Scinco). The dynamic light scattering (DLS) measurement was performed using a Nano-ZS instrument (Malvern). Transmission electron microscopy (TEM) images were captured with an electron microscope (JEOL-2100F) operating at 200 kV. Fourier transform infrared (FT-IR) spectra were recorded on a VERTEX 80/80v FT-IR spectrometer (BRUKER). All confocal laser scanning microscopy images were obtained using an Olympus Fluoview FV1200 confocal laser scanning microscope.

1.2 Synthesis of MPa-TEG (MT) (R=-$OCH_2CH_2OCH_2CH2$-OCH_2CH_2OH)

Methylpheophorbide a (MPa) (0.1 g, 0.16 mmol) and 4-(dimethylamino) pyridine (0.016 g, 0.13 mmol) were dissolved in toluene. Then, tri(ethylene glycol) (0.033 mL, 0.25 mmol) was added, and the whole mixture was heated for 16 hrs. The mixture was cooled to room temperature and treated with aqueous ammonium chloride. Next, the mixture was extracted twice with methylene chloride. The organic layer was washed with water, and the solvent was evaporated. The residual solid was purified by gradient column chromatography on silica. The fast moving, nonpolar side products were separated first by eluting the column with CH_2Cl_2/EtOAc = 19/1. Then, the desired product (MT) was separated by eluting the column with EtOAc/THF = 9/1. The black purple product was obtained by recrystallization from CH_2Cl_2/hexanes, yielding 0.073 g (61%).

1.3 Preparation of nanocomplexes

MT (10 mM in DMSO) and EGCG (10 mM in water) were first prepared as stock solutions. Then, 10, 30, and 50 μL of the EGCG stock solution were added to 980, 960 and 940 μL of water, respectively. Finally, 10 μL of the MT stock solution were added under ultrasonic stirring. Uniformly dispersed nanocomplexes were obtained after overnight oscillation.

1.4 Cell culture

Human cervical adenocarcinoma HeLa cells were purchased from the Korean Cell Line Bank (Seoul, Korea) and cultured in EMEM (Eagle's Minimum Essential Medium containing NEAA) supplemented with 10% fetal bovine serum, 100 U/ml penicillin and 100 U/ml streptomycin at 37 °C in a humidified atmosphere containing 5% CO_2.

1.5 Cellular uptake and colocalization

Cellular uptake. Cells were seeded in a 35 mm confocal dish and incubated for 24 h. Then, the medium was replaced with 1 mL of medium containing MTE (5 μM) after washes with DPBS. Cell images were captured with a confocal laser scanning microscope after different incubation times.

Colocalization assay. Cells were seeded in a 35 mm confocal dish and incubated for 24 h. Then, the medium was replaced with 1 mL of medium containing MTE (5 μM) after washes with DPBS and cultured for 2 h. LysoTracker Green DND 26 and MitoTracker Green FM (500 nM) were utilized to stain the cells separately for 30 min. Cell images were captured with a confocal laser scanning microscope.

1.6 Determination of Cytotoxicity

MTT assay. A total of 3 X 10^4 cells were seeded in each well of a 96-well plate with 100 μL of culture media and then incubated for 24 h. Then, 100 μL of new culture media containing different concentrations of the samples were added to the plate after washes with DPBS and cultured for an additional 2 h. For the untreated group, the medium was removed, and 90 μL of fresh MEM together with 10 μL of MTT solution (5 mg/mL) were added to each well and cultured for an additional 4 h. For the treatment group, the medium containing samples was replaced with fresh medium for light or water bath treatment and then cultured for 24 h. Finally, the medium was removed again, 100 μL of DMSO were added to each well. The blue formazan crystals were fully dissolved by shaking at low speed for 10 min. The absorbance of each well was measured at OD 490 nm with a SpectraMax Microwell plate reader. The viability of the cells that were not incubated with samples or treated with heat or light was set to 100%. Data are presented as the means and standard deviations (SD) of three independent experiments.

Fluorescence microscopy. Cells were seeded in 35-mm glass-bottomed dishes at a density of 3 X 10^5 cells per dish in culture media. After 24 hrs, the cells were incubated with 3 μM sample for 2 hrs and treated with 45 °C for 30 min or a 690 nm laser (0.1 W/cm^2) and incubated for 16 hrs before staining with calcein-AM and propidium iodide, and fluorescence images were acquired using a confocal microscope (FV1200, Olympus, Japan).

Flow cytometry. The levels of apoptotic cells after treatment were evaluated using an Annexin V-FITC Apoptosis Detection Kit with 7-AAD (7-amino-actinomycin D) (Biolegend, CA, USA). Cells were cultured for 24 hrs in 6-well plates, incubated with 3 μM sample for 2 hrs and treated at 45 °C for 30 min or a 690 nm laser (0.1 W/cm^2) and incubated for 16 hrs. Cells were trypsinized, washed twice with cold PBS, resuspended in Annexin V Binding Buffer containing Annexin V-FITC/7-AAD, incubated for 15 min at room temperature in the dark and analyzed using a BD LSRFortessa (Becton Dickinson). A total of 10,000 cells were used for each analysis, and experiments were performed in triplicate.

1.7 Determination of reactive oxygen species (ROS)-scavenging activity

Fluorescence microscopy. Cells were seeded in 35-mm glass-bottomed dishes at a density of 3 X 10^5 cells per dish in culture media. After 24 hrs, HeLa cells were preincubated with 20 μM MTE for 2 hrs, treated with 500 μM H_2O_2 or 100 μM antimycin A for 30 min and stained with 5 μM DCFHDA for 30 min. Then, fluorescence images were acquired by confocal microscopy.

Flow cytometry. Cells were cultured for 24 hrs in 6-well plates, preincubated with 20 μM MTE for 2 hrs, treated with 500 μM H_2O_2 or 100 μM antimycin A for 30 min, and stained with 5 μM DCFHDA for 30 min. Cells were trypsinized, washed twice with cold PBS, resuspended in PBS and analyzed using a BD LSRFortessa flow cytometer (Becton Dickinson). A total of 10,000 cells were used for each analysis, and experiments were performed in triplicate.

1.8 Western blotting

HeLa cells were incubated with 3 μM sample for 2 hrs and treated at 45 °C for 30 min or with a 690 nm laser (0.1 W/cm^2) and incubated for 16 hrs. The protein lysate was harvested with RIPA buffer (50 mM Tris-HCl, 150 mM NaCl, 1% Triton X-100, 1% sodium deoxycholate, 0.1% SDS, and 1 mM EDTA, pH

7.4). Proteins were incubated for 30 min on ice and centrifuged at 13,000 rpm for 10 min at 4 °C. Supernatants were collected, and protein concentrations were quantified using a BCA protein assay kit (Thermo Scientific). Equivalent amounts of protein (35 μg) were separated on SDS–PAGE gels and transferred to PVDF (polyvinylidene difluoride) membranes. The membranes were blocked for 1 hour with blocking buffer (5% nonfat dry milk in TTBS (0.1% Tween 20-TBS)), washed with TTBS, and incubated with the primary antibody diluted in blocking buffer overnight at 4 °C. The membranes were then washed with TBST three times, incubated with secondary antibody for 2 hrs and detected using Pierce ECL Western blotting substrate (Thermo Scientific). Primary antibodies against HSP70, HSP90, PARP, caspase-3, and β-actin were purchased from Santa Cruz Biotechnology, and the secondary HRP-conjugated anti-mouse IgG antibody was purchased from Cell Signaling Technology.

1.9 Statistical analysis

All statistical analyses were performed using Excel software. Data are presented as the means ± SD of measurements of n experiments conducted using different assays. Statistical significance was assessed using an independent-samples t test. A P value < 0.05 was considered statistically significant.

2. Supporting Figures

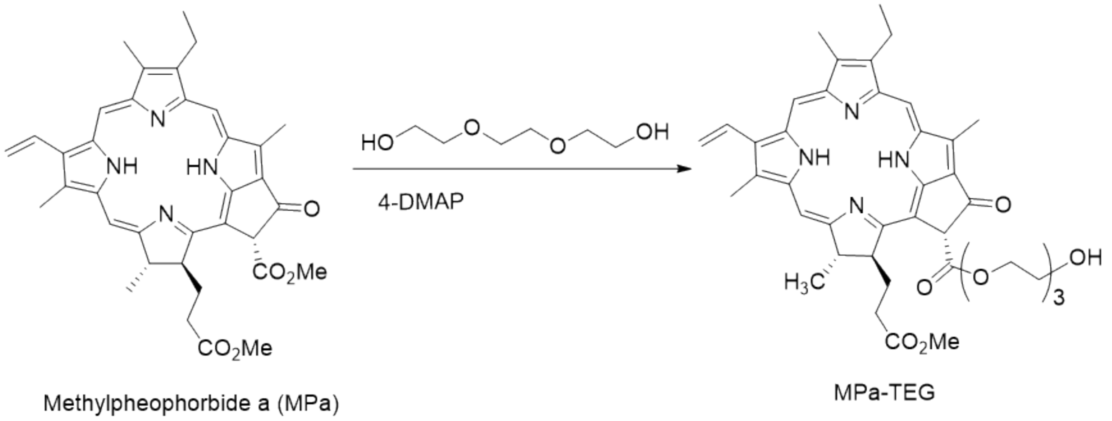

Scheme S1. Synthesis of compound MPa-TEG (MT).

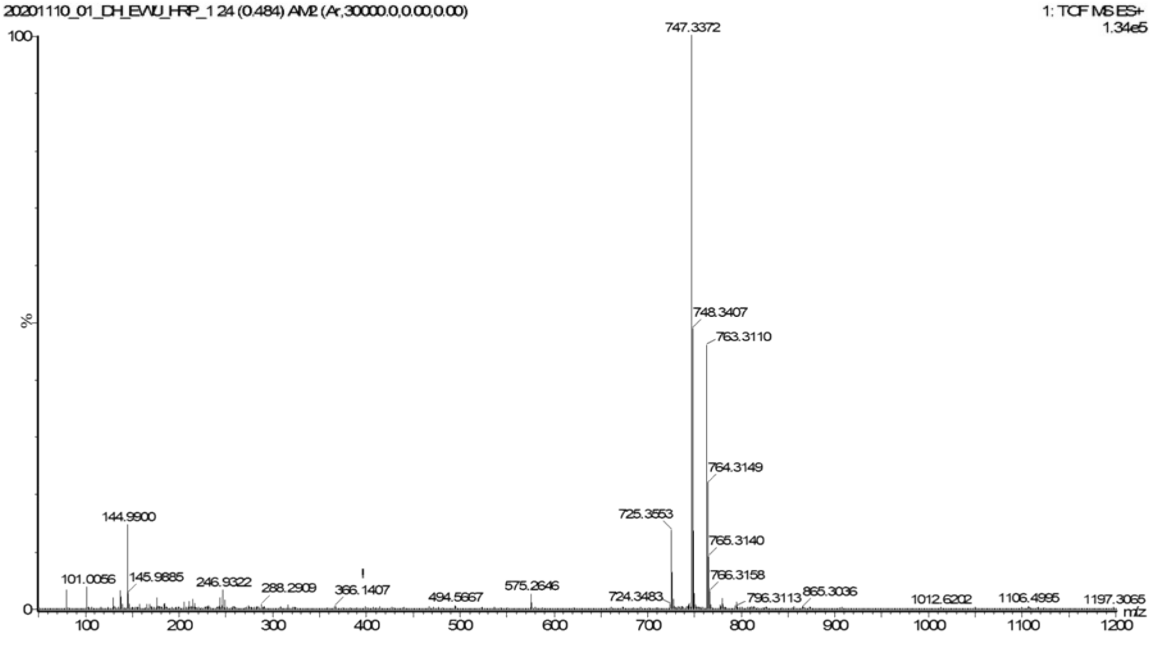

Fig. S1. HR-ESI-MS of MT (C41H48N4O8Na+, MS calcd for 747.3364, found 747.3372).

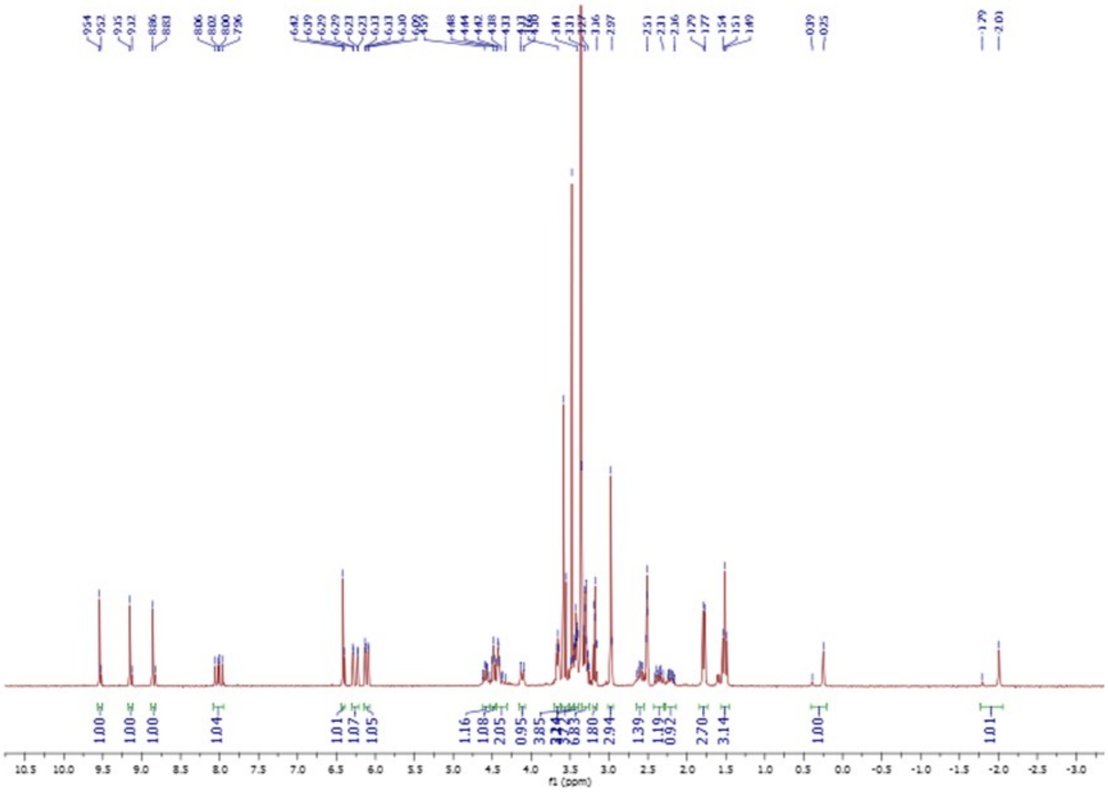

Fig. S2. ^{1}H-NMR spectrum of MT in DMSO-d_6.

^{1}H NMR (300 MHz, DMSO-d_6) δ: 9.53 (d, J = 6.7 Hz, 1H), 9.14 (d, J = 8.7 Hz, 1H), 8.84 (d, J = 9.9 Hz, 1H), 8.01 (dd, J = 17.8, 11.4 Hz, 1H), 6.41 (d, J = 7.1 Hz, 1H), 6.26 (dd, J = 17.8, 1.6 Hz, 1H), 6.11 (dd, J = 11.6, 1.5 Hz, 1H), 4.58 (dt, J = 10.7, 8.0 Hz, 1H), 4.48 (t, J = 5.5 Hz, 1H), 4.45 – 4.31 (m, 2H), 4.11 (d, J = 9.5 Hz, 1H), 3.70 – 3.63 (m, 2H), 3.57 (d, J = 9.6 Hz, 4H), 3.50 – 3.45 (m, 4H), 3.45 – 3.39 (m, 3H), 3.35 – 3.24 (m, 7H), 3.20 – 3.16 (m, 2H), 2.97 (d, J = 3.3 Hz, 3H), 2.66 – 2.55 (m, 1H), 2.36 (dt, J = 14.7, 7.6 Hz, 1H), 2.28 – 2.15 (m, 1H), 1.78 (d, J = 7.2 Hz, 3H), 1.51 (t, J = 7.5 Hz, 3H), 0.32 (d, J = 42.2 Hz, 1H), -1.90 (d, J = 63.9 Hz, 1H).

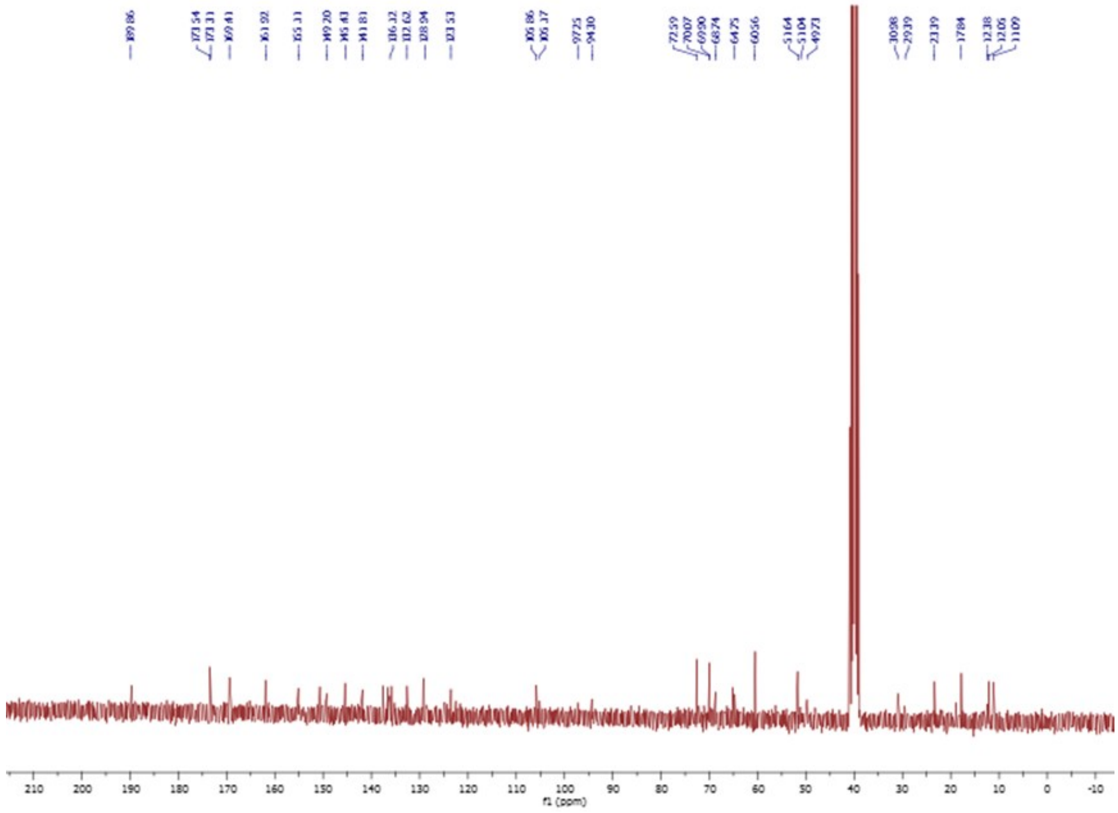

Fig. S3. ^{13}C-NMR spectrum of MT in DMSO-d_6.

^{13}C NMR (75 MHz, DMSO-d_6) δ: 189.86, 173.54, 173.31, 169.41, 161.92, 155.11, 150.67, 149.20, 145.43, 141.81, 137.60, 136.62, 136.12, 135.82, 135. 14, 132.62, 129.25, 129.12, 128.94, 123.53, 105.86, 105.17, 97.25, 94.30, 72.59, 70.07, 69.90, 68.74, 65.14, 64.75, 60.56, 51.64, 51.04, 49.73, 30.98, 29.39, 23.39, 18.90, 17.84, 12.38, 12.05, 11.09.

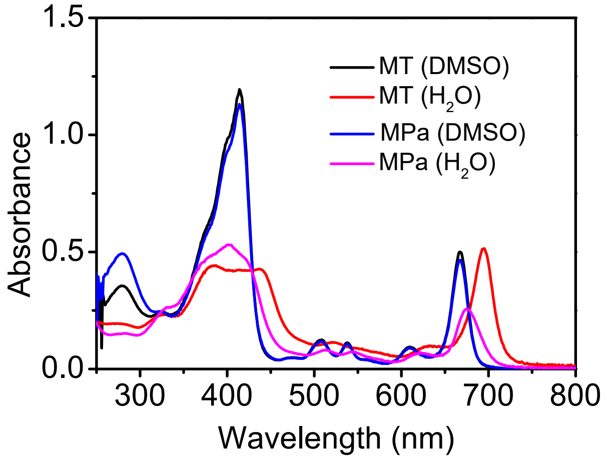

Fig. S4. Electronic absorption spectra of MT and MPa (10 μM) in DMSO and H_2O, respectively.

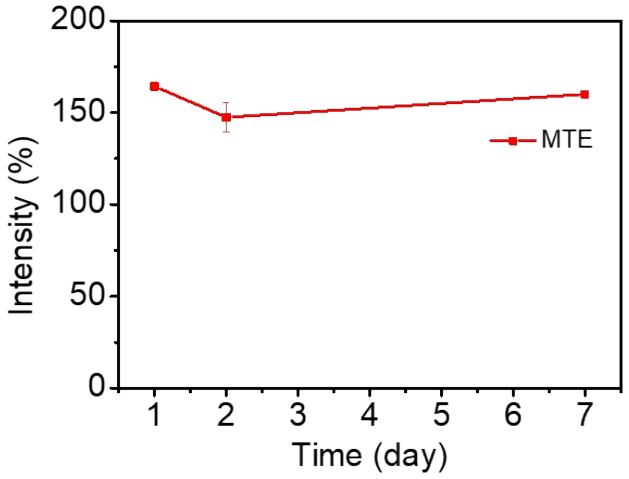

Fig. S5. The stability of MTE over time.

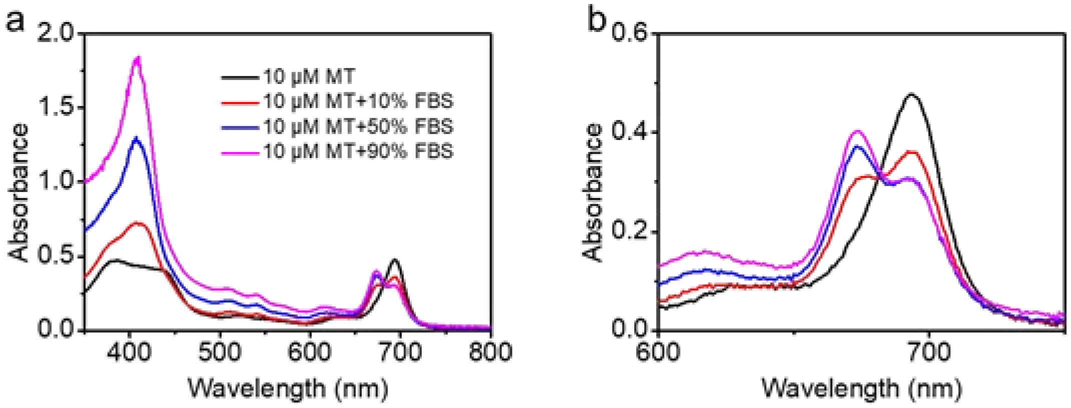

Fig. S6. The stability of J-aggregate under physiological conditions. (a) Electronic absorption of MT incubating with different concentrations of FBS. (b) Electronic absorption from 600 nm to 750 nm.

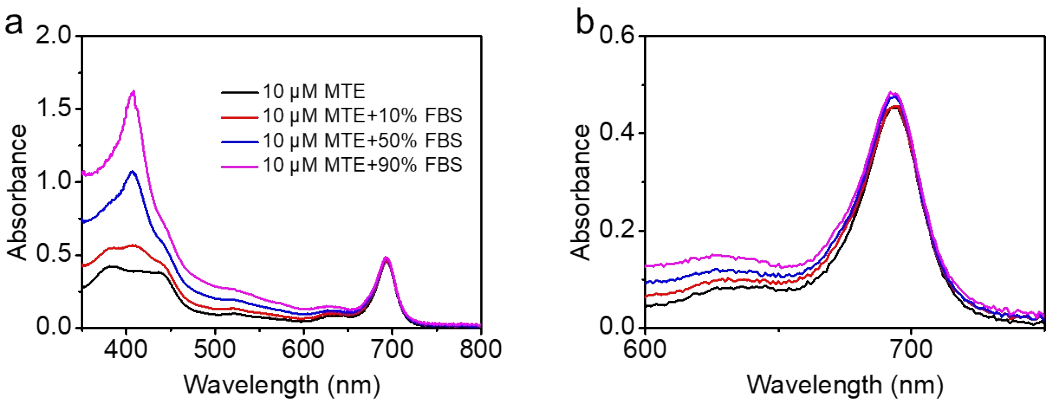

Fig. S7. The stability of J-aggregate under physiological conditions. (a) Electronic absorption of MTE incubating with different concentrations of FBS. (b) Electronic absorption from 600 nm to 750 nm.

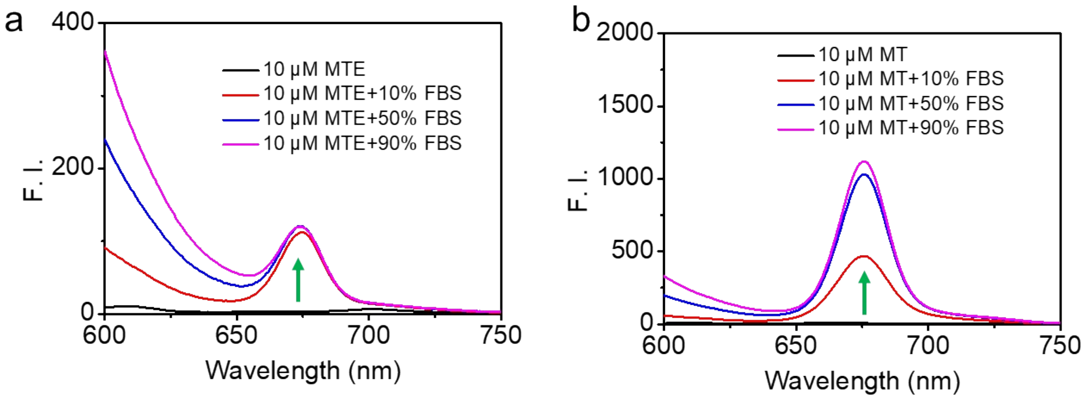

Fig. S8. The intelligently enhanced fluorescence by FBS. (a) MTE. (b) MT.

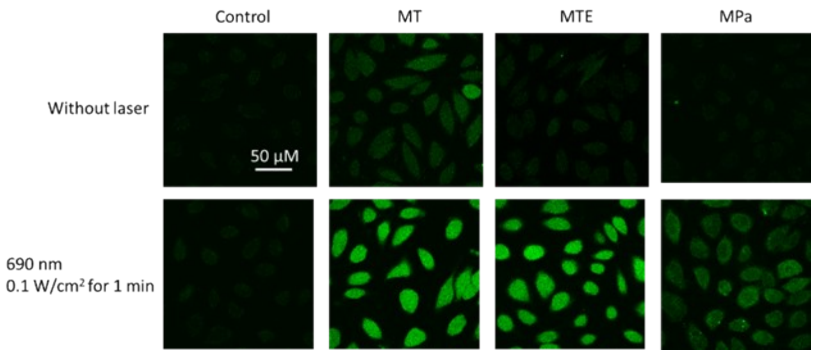

Fig. S9. ROS generation using DCFHDA as the fluorescent probe in Hela cells.

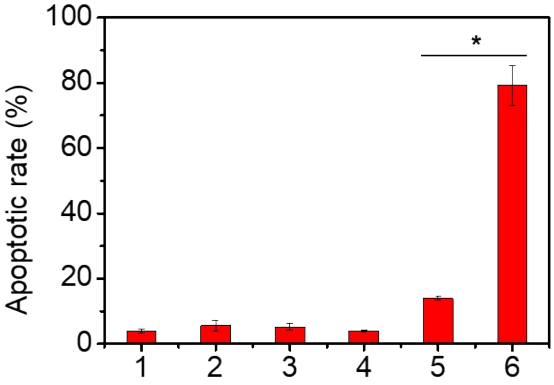

Fig. S10. Quantitative analysis of the percentage of apoptotic cells (early + late apoptosis). Data are presented as the means ± standard deviation from three independent experiments. *, P<0.05, significantly different from control. (1. control, 2. 45 ℃, 30 min, 3. 690 nm laser, 4. MTE, 5. MTE + 45 ℃, 30 min, 6. MTE + 690 nm laser).

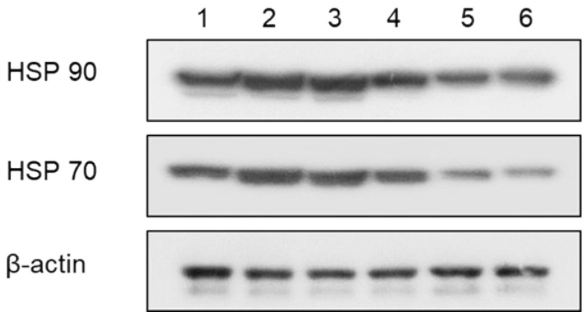

Fig. S11. Protein expression level of HSP 70, HSP 90 in HeLa cells. (1. control, 2. 45 ℃, 30 min, 3. 690 nm laser, 4. MTE, 5. MTE + 45 ℃, 30 min, 6. MTE + 690 nm laser).

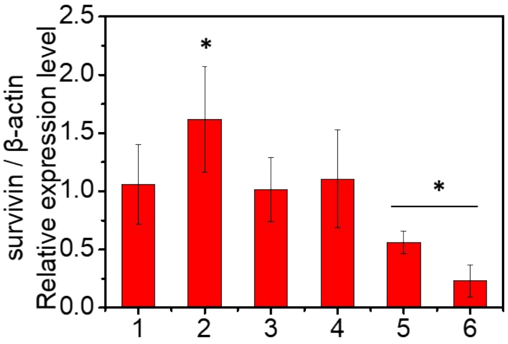

Fig. S12. Bar graph of relative protein expression level of survivin from three independent experiments (n = 3) were calculated with Image J. Expression of β-actin was taken as a loading control. *, P<0.05, significantly different from control. (1. control, 2. 45 ℃, 30 min, 3. 690 nm laser, 4. MTE, 5. MTE + 45 ℃, 30 min, 6. MTE + 690 nm laser).

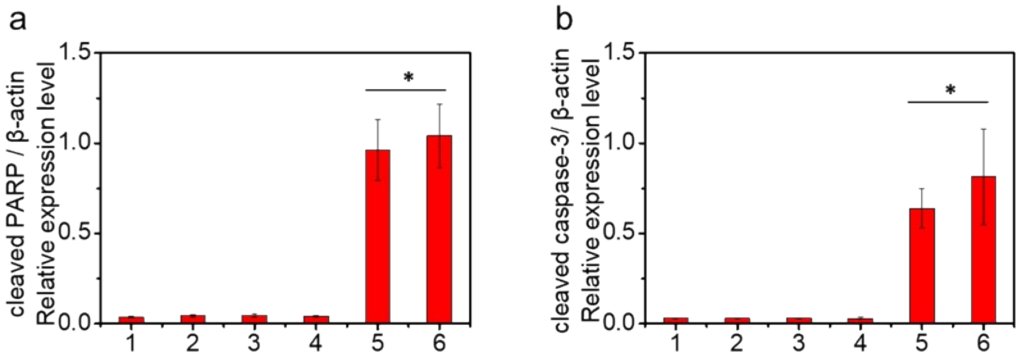

Fig. S13. (a) Protein expression level of cleaved PARP and cleaved caspase 3 in HeLa cells. (b) Bar graph of the relative protein expression level of cleaved PARP and cleaved caspase 3 from three independent experiments (n = 3) were calculated with Image J. Expression of β-actin was taken as a loading control. *, P<0.05, significantly different from control. (1. control, 2. 45 ℃, 30 min, 3. 690 nm laser, 4. MTE, 5. MTE + 45 ℃, 30 min, 6. MTE + 690 nm laser).

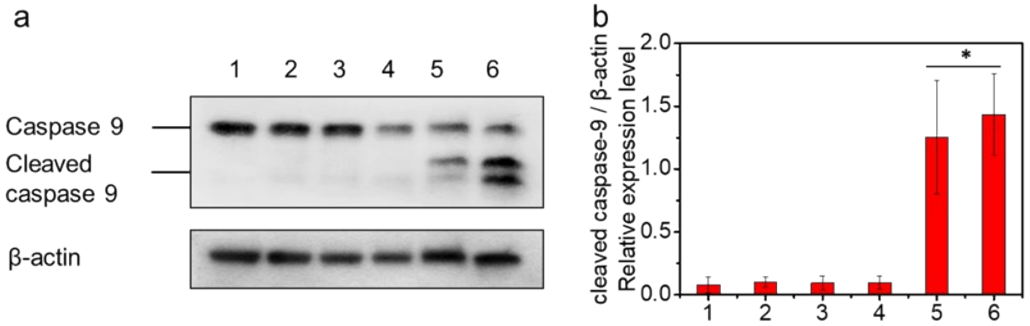

Fig. S14. (a) The protein expression level of cleaved caspase 9 in HeLa cells. (b) Bar graph of the relative protein expression level of cleaved caspase 9 from three independent experiments (n = 3) were calculated with Image J. Expression of β-actin was taken as a loading control. *, P<0.05, significantly different from control. (1. control, 2. 45 ℃, 30 min, 3. 690 nm laser, 4. MTE, 5. MTE + 45 ℃, 30 min, 6. MTE + 690 nm laser).
